# The 21-gene recurrence score in early non-ductal breast cancer: a National Cancer Database analysis

**DOI:** 10.1038/s41523-021-00368-9

**Published:** 2022-01-13

**Authors:** Della Makower, Jiyue Qin, Juan Lin, Xiaonan Xue, Joseph A. Sparano

**Affiliations:** 1 Montefiore Einstein Center for Cancer Care, New York, NY USA; 2 Albert Einstein Cancer Center, Bronx, NY USA

**Keywords:** Breast cancer, Cancer genomics

## Abstract

The 21-gene recurrence score (RS) is prognostic for recurrence and predictive of chemotherapy benefit in early estrogen receptor-positive (ER +) HER2-negative (HER2-) breast cancer (BCA). We evaluated clinicopathologic characteristics, RS and chemotherapy benefit in invasive ductal carcinoma (IDC), invasive lobular carcinoma (ILC), and carcinomas of mixed histologies (ductal + lobular (DLC), ductal + other (DOC), lobular + other (LOC)). Women diagnosed between 1/1/2010 and 1/1/2014 with ER + HER2- BCA, measuring <5 cm, with 0–3 involved axillary nodes, surgery as first treatment, and available RS, were identified from the NCDB. Associations between categorical variables were examined using chi-square test. Cox proportional hazards model was used to examine overall survival (OS) differences among histology subtypes. IDC was associated with smaller size, high grade, and RS > 26. ILC was associated with larger size, and least likely to be high grade (*p* < 0.0001). Lobular histology was associated with lower incidence of RS > 26. IDC patients (pts) were more likely to receive chemotherapy than pts with other histologies (*p* < 0.0001). OS for IDC, ILC and DOC were similar. DLC was associated with improved OS (HR 0.82, *p* = 0.02). Adjuvant chemotherapy was associated with improved OS in IDC (HR = 0.76, *p* < 0.0001) but not in ILC (HR = 0.99, *p* = 0.93), DLC (HR = 1.04, *p* = 0.86), DOC (HR = 0.87, *p* = 0.71), or LOC (HR = 2.91, *p* = 0.10). Lobular and mixed BCA histologies have distinct clinicopathologic features compared with IDC, and are less likely to have high RS. OS is similar for IDC and ILC. Although chemotherapy benefit was seen only in IDC, benefit for ILC with RS > 26 cannot be excluded.

## Introduction

Breast cancer is a heterogenous disease, which includes several histologic morphologies^1^. Invasive ductal carcinoma (IDC), the most common form of breast cancer (BCA), comprises ~80% of cases. Invasive lobular carcinoma (ILC), the second most prevalent BCA histology, represents about 10–15% of cases^2,3^. ILC is characterized by E-cadherin loss, and is typically hormone receptor-positive and HER2-negative^2,4^. Early ILC is more difficult to detect mammographically than IDC^5^, and advanced ILC is associated with a predilection for metastases to the peritoneum, gastrointestinal tract, and meninges^4,6,7^. Despite these differences, IDC and ILC are typically managed similarly.

The 21-gene Oncotype DX gene expression assay is prognostic for recurrence and predictive of chemotherapy benefit in early HR + HER2-negative BCA^8–12^. The TAILORx and RxPONDER trials established that postmenopausal women with HR + BCA and Oncotype DX recurrence score (RS) < 25 involving up to three axillary nodes do not benefit from chemotherapy, whereas premenopausal women with positive axillary nodes or with negative nodes and RS 16–25 may derive some chemotherapy benefit that has been ascribed to chemotherapy-induced amenorrhea^8,13^. ILC is typically associated with low to intermediate RS^4,14–16^, and with poorer response to chemotherapy than IDC^17–19^. We sought to evaluate differences in clinicopathologic characteristics, RS and chemotherapy benefit between IDC, ILC, and carcinomas of mixed histologies.

## Results

### Patient characteristics

**Fig. 1:**
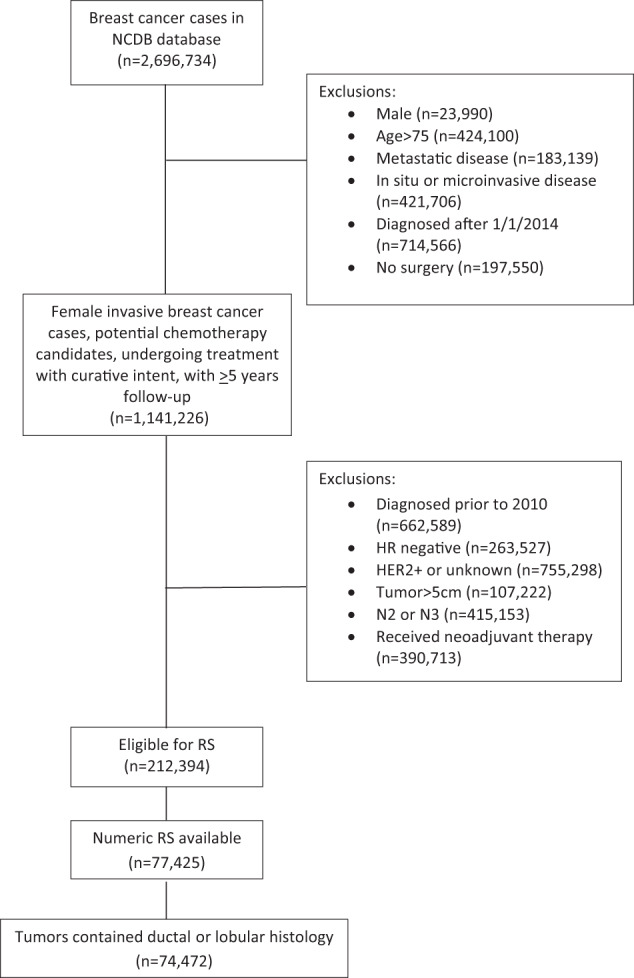
CONSORT diagram for patient selection. NCDB National Cancer Database, HR hormone receptor, N2, 4–9 involved axillary nodes, N3 10 or more involved axillary nodes, RS 21-gene recurrence score.

As shown in Fig. 1, of 2,696,734 women with breast cancer in the NCDB database, 74,472 patients met inclusion criteria for this analysis; 62,395 (83.8%) node negative (N0) and 12,077 (16.2%) with up to three involved axillary nodes (N1). 57,615 patients (77.4%) had IDC; 8693 (11.7%) ILC; 5393 (7.2%) DLC; 2459 (3.3%) DOC; and 312 (0.4%) LOC.

### Associations between tumor subtypes and clinicopathologic characteristics

Associations between tumor subtypes and clinicopathologic characteristics are summarized in Table 1. Though statistically significant, variations in median age, race, and ethnicity among tumor subtypes were small in magnitude. DOC and LOC were slightly more common among Black women than other tumor subtypes, and DOC was slightly more common in Hispanics. IDC was associated with smaller tumor size. 79.2% of IDC measured 20 mm or less, compared with 65.5% of ILC, 71.2% of DLC, 74.7% DOC, and 70.4% LOC (*p* < 0.0001). IDC was also most likely to be high grade, while ILC was least. 18% of IDC were high grade, compared with 5.3% of ILC, 11.0% of DLC, 10.2% DOC, and 11.2% LOC (*p* < 0.0001).

**Table 1:** Patient characteristics.

Characteristic	Tumor subtype					*P* value
	IDC (*n* = 57,615)	ILC (*n* = 8693)	DLC (*n* = 5393)	DOC (*n* = 2459)	LOC (*n* = 312)	
Age, median (IQR)	58 (50, 65)	60 (52, 66)	59 (51, 65)	58 (50, 66)	60 (52, 66)	*p* < 0.0001
Race						
White	50,549 (87.7)	7816 (89.9)	4794 (88.9)	2091 (85)	271 (86.9)	*p* < 0.0001
Black	4241 (7.4)	618 (7.1)	330 (6.1)	235 (9.6)	30 (9.6)	
Other	2403 (4.2)	194 (2.2)	218 (4.0)	114 (4.6)	10 (3.2)	
Unknown	422 (0.7)	65 (0.7)	51 (0.9)	19 (0.8)	1 (0.3)	
Ethnicity						*p* = 0.0014
Non-Hispanic	53,416 (92.7)	8131 (93.5)	5022 (93.1)	2260 (91.9)	294 (94.2)	
Hispanic	2055 (3.6)	276 (3.2)	213 (3.9)	112 (4.6)	4 (1.3)	
Unknown	2144 (3.7)	286 (3.3)	158 (2.9)	87 (3.5)	14 (4.5)	
Tumor size (mm)						*p* < 0.0001
0–20	45,630 (79.2)	5693 (65.5)	3839 (71.2)	1838 (74.7)	220 (70.5)	
21–50	11,985 (20.8)	3000 (34.5)	1554 (28.8)	621 (25.3)	92 (29.5)	
Grade						*p* < 0.0001
1	15,366 (26.7)	2176 (25.0)	1081 (20.0)	1021 (41.5)	121 (38.8)	
2	29,243 (50.8)	5176 (59.5)	3447 (63.9)	1086 (44.2)	135 (43.3)	
3	10,392 (18.0)	462 (5.3)	594 (11.0)	251 (10.2)	35 (11.2)	
Unknown	2614 (4.5)	879 (10.1)	271 (5.0)	101 (4.1)	21 (6.7)	
Node involvement						*p* < 0.0001
0	48,268 (83.8)	7341 (84.4)	4398 (81.6)	2133 (86.7)	255 (81.7)	
1–3	9347 (16.2)	1352 (15.6)	995 (18.4)	326 (13.3)	57 (18.3)	
Surgical procedure						*p* < 0.0001
Lumpectomy	39,481 (68.5)	4817 (55.4)	3125 (57.9)	1564 (63.6)	173(55.4)	
Mastectomy	18,131 (31.5)	3875 (44.6)	2266 (42.0)	894 (36.4)	139 (44.6)	
Chemotherapy						*p* < 0.0001
Yes	15,773 (27.4)	1680 (19.3)	1180 (21.9)	505 (20.5)	60 (19.2)	
No	41,069 (71.3)	6880 (79.1)	4129 (76.6)	1917 (78)	245 (78.5)	
Unknown	773 (1.3)	133 (1.5)	84 (1.6)	37 (1.5)	7 (2.2)	
Hormonal therapy						*p* < 0.0001
Yes	56,351 (97.8)	8564 (98.5)	5323 (98.7)	2412 (98.1)	308 (98.7)	
No	1000 (1.7)	101 (1.2)	56 (1.0)	38 (1.5)	3 (1.0)	
Unknown	264 (0.5)	28 (0.3)	14 (0.3)	9 (0.4)	1 (0.3)	

### Associations between tumor subtypes and RS

**Fig. 2:**
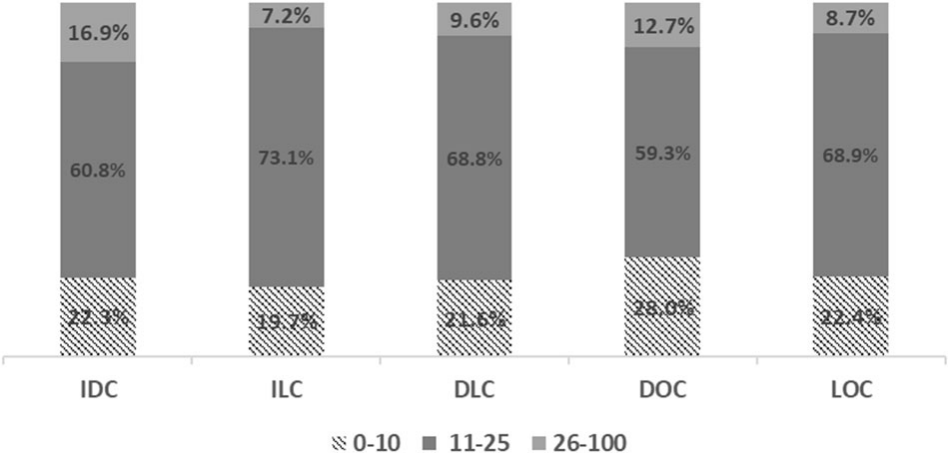
Distribution of RS among breast cancer histologic subtypes. Distribution of low (0–10), intermediate (11–25), and high (26–100) RS in IDC, ILC, DLC, DOC, and LOC (*p* < 0.0001).

Associations between tumor subtypes and RS are shown in Fig. 2. Significant differences in RS distribution among tumor subtypes were seen. Lobular histology was associated with a lower incidence of high RS (>26), with rates of high RS of 7.2% and 8.7% for ILC and LOC, respectively, compared with 16.9% for IDC and 12.7% for DOC (*p* < 0.0001). Tumors containing both ductal and lobular features (DLC) had a 9.6% incidence of RS > 26, somewhat higher than seen in ILC and LOC, but lower than rates for IDC and DOC. DOC was associated with the highest likelihood of low RS of 0–10, with 28% of DOC having RS 0-10, compared with 22.3%, 19.7%, 21.6%, and 22.4%, for IDC, ILC, DLC, and LOC, respectively (*p* < 0.0001). Among the tumor subtypes, ILC was least likely to have both high and low RS.

### Associations between tumor subtypes and cancer therapy

Associations between tumor subtypes and treatment are also summarized in Table 1. Presence of lobular histology was associated with increased likelihood of mastectomy compared to breast conserving surgery. 44.6% of patients with ILC and 44.6% of patients with LOC required mastectomy, compared with 31.5% of IDC and 36.4% of DOC. Again, DLC exhibited a mastectomy rate that was intermediate between IDC and ILC, with 42.0% of IDC patients undergoing mastectomy.

**Fig. 3:**
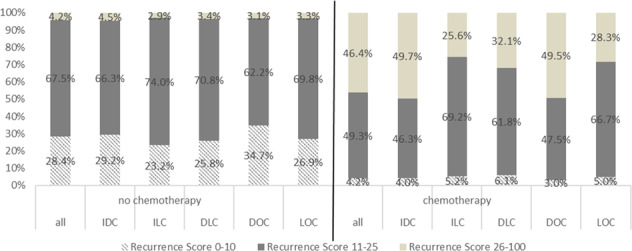
Association of adjuvant chemotherapy use with RS. Association of adjuvant chemotherapy use with low (0–10), intermediate (11–25, and high (26–100) RS for entire patient cohort (no chemotherapy: *n* = 54,240; chemotherapy: *n* = 19,198) and for each histologic subtype. (IDC: no chemotherapy: *n* = 41,069; chemotherapy: *n* = 15,773; ILC: no chemotherapy: *n* = 6880; chemotherapy: *n* = 1680; DLC: no chemotherapy: *n* = 4129; chemotherapy: *n* = 1180; DOC: no chemotherapy: *n* = 1917; chemotherapy: *n* = 505; LOC: no chemotherapy: *n* = 245; chemotherapy: *n* = 60).

IDC was associated with increased likelihood of use of adjuvant chemotherapy. 27.4% of IDC patients received chemotherapy, compared with 19.3%, 21.9%, 20.5% and 19.2% for ILC, DLC, DOC, and LOC, respectively (*p* < 0.0001). Chemotherapy use was highly associated with high RS in the full patient cohort (*p* < 0.0001) (Fig. 3).

### Associations between tumor subtypes and OS

**Fig. 4:**
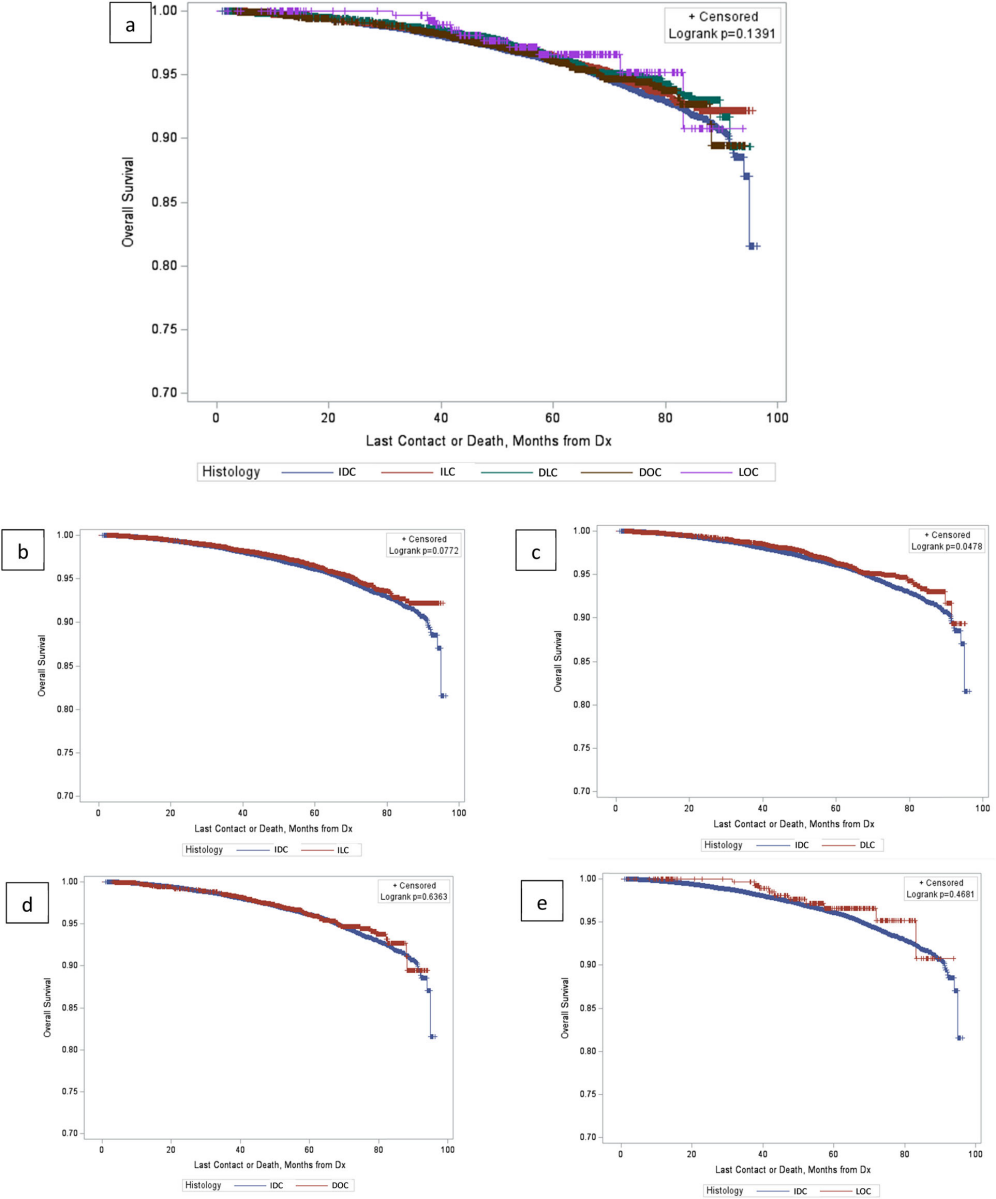
Kaplan–Meier curve for OS in different histological subtypes. Kaplan–Meier curve for OS comparing different tumor histologic subtypes. (**a**: all histologic subtypes, *p* = 0.1391; **b**: IDC vs ILC, *p* = 0.0772; **c**: IDC vs DLC, *p* = 0.0478; **d**: IDC vs DOC, *p* = 0.6363; **e**: IDC vs LOC, *p* = 0.4681).

When compared with IDC, overall survival (OS) (adjusted for age, race, ethnicity, rurality, median income, and educational level of area of residence, RS, tumor size, grade, node involvement, Charlson-Deyo comorbidity index, and treatment) for ILC was similar (HR 0.911; 95% CI 0.798, 1.039; *p* = 0.165). OS for DOC and LOC were also similar to IDC (HR 1.002; 95% CI 0.800, 1.254; *p* = 0.989 for DOC, and HR 0.530; 95% CI 0.237, 1.181; *p* = 0.120). In contrast, DLC had improved OS, compared with IDC (HR 0.824; 95% CI 0.696, 0.974; *p* = 0.024). Kaplan-Meier curves for OS in each histologic subtype are shown in Fig. 4.

#### Factors associated with OS in tumor subtypes

Factors associated with poorer OS in the full patient cohort included larger tumor size, node involvement, high grade disease, high RS, increasing age, Black race, and increasing comorbidity index (all *p* < 0.0001). These factors were also all significantly associated with poorer OS in IDC (all *p* < 0.0001). High RS (>26) remained prognostic for poorer OS in ILC (HR 1.906; 95% CI 1.151, 3.157; *p* = 0.0122), and in DLC (HR 2.557; 95% CI 1.398, 4.676); *p* = 0.0023), but not in DOC (HR 1.385; 95% CI 0.545, 3.519; *p* = 0.4942). Of note, a full analysis could not be performed on LOC, due to small sample size (*n* = 312, with 10 deaths). Other factors associated with poorer OS in ILC included larger tumor size (*p* = 0.0188), N1 disease (*p* = 0.0002), increasing age (*p* < 0.0001), and increasing comorbidity index (*p* < 0.0001). Black race and high grade disease were not associated with OS in ILC. For DLC, factors associated with poorer OS, apart from high RS, included increasing age (*p* < 0.0001), higher Charlson-Deyo comorbidity index (*p* = 0.0081) and Black race (*p* = 0.0025). Tumor size, grade, and node involvement were not associated with OS in DLC.

### Evaluation of chemotherapy use and chemotherapy benefit

Receipt of adjuvant chemotherapy was associated with improvement in OS in the full patient cohort (HR 0.800, 95% CI 0.716, 0.894, *p* = 0.0001). IDC patients were more likely to receive chemotherapy than patients with other tumor histologies. 27.4% of IDC patients received adjuvant chemotherapy, compared with 19.3%, 21.9%, 20.5% and 19.2% for ILC, DLC, DOC, and LOC, respectively (*p* < 0.0001). Chemotherapy use was associated with increasing RS in the full patient cohort and in all tumor subtypes (all *p* < 0.0001, Fig. 3), although patients with ILC and RS 11–25 were more likely to receive chemotherapy than were patients with other tumor histologies. Chemotherapy use was also associated with larger tumor size, higher grade, N1 disease, Black race, and lower comorbidity score in the full patient cohort and in IDC (all *p* < 0.0001). Significant associations with chemotherapy use persisted for tumor size and nodal involvement for all tumor subtypes (*p* < 0.0001) except LOC. High grade disease was significantly associated with chemotherapy use in all tumor subtypes (*p* < 0.0001 for IDC, ILC, DLC and DOC, and 0.012 for LOC).

**Fig. 5:**
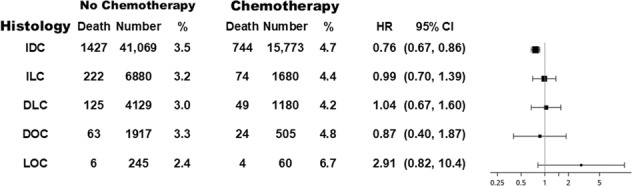
Chemotherapy benefit in different histological subtypes. Evaluation of overall survival benefit from adjuvant chemotherapy in different tumor histologic subtypes (adjusted for age, race, ethnicity, rurality, median income and educational level of area of residence, RS, tumor size, grade, node involvement, and Charlson-Deyo comorbidity index).

Evaluation of chemotherapy benefit in different tumor subtypes demonstrated that receipt of adjuvant chemotherapy was associated with improved OS in IDC (HR = 0.76; 95% CI 0.672, 0.864; *p* < 0.0001). In contrast, adjuvant chemotherapy was not associated with improvement in OS in ILC (HR = 0.986; 95% CI 0.700, 1.389; *p* = 0.9349), DLC (HR 1.039; 95% CI 0.673, 1.605; *p* = 0.8626), DOC (HR = 0.866; 95% CI 0.401, 1.871; *p* = 0.7148) and LOC (HR = 2.909; 95% CI 0.816, 10.372; *p* = 0.0996) (Fig. 5). However, because IDC is the largest subtype, the lack of statistical significance associated with chemotherapy for other subtypes may result from lack of statistical power (The minimum detectable HR associated with chemotherapy for ILC, DLC, DOC, and LOC is 0.80, 0.75, 0.63, 0.11, correspondingly, with 80% power and two-sided type I error rate of 5%.).

## Discussion

Although IDC and ILC are the most common BCA subtypes, IDC, represents ~80% of cases. The majority of clinical trials which inform BCA management do not distinguish between BCA subtypes, and their findings are likely driven by the behavior of IDC. We therefore sought to evaluate differences in clinicopathologic characteristics, RS distribution, and chemotherapy benefit between early HR + HER2-negative IDC and other HR + HER2-negative BCA subtypes in the NCDB database. We found statistically significant and clinically relevant differences in clinicopathologic features, RS distribution, and chemotherapy benefit between IDC, ILC and mixed ductal and lobular histologies.

As previously reported by other investigators^2,3^, we found that ILC was associated with larger tumors, lower histologic grade, and with increased use of mastectomy and decreased use of chemotherapy compared with IDC, but with similar OS. We also found improved adjusted OS in patients with DLC compared with IDC, which has not been previously reported.

We found a lower incidence of high RS in tumors with lobular histology compared with IDC. ILC was also associated with a lower incidence of low RS, and was therefore most likely to have intermediate RS of 11–25. High RS was prognostic for OS in the full patient cohort, and in IDC, and remained prognostic for OS in ILC and DLC, but not in DOC. In addition, we found that the OS benefit of adjuvant chemotherapy was limited to patients with IDC.

Several other investigators have evaluated RS in in non-ductal BCA histologies, and our findings complement their work. Tadros et al. evaluated RS in 610,350 tumor specimens from Genomic Health’s clinical laboratory, and found that ILC, DLC, DOC, and LOC all had significantly lower mean RS compared with IDC, but did not evaluate the impact of RS on prognosis or chemotherapy benefit in non-ductal histologies^14^. Christgen et al. compared RS and clinicopathologic prognostic factors in patients with node positive (N+) and high risk N0 ER+, HER2-negative lobular (*n* = 353) and non-lobular (*n* = 2232) BCA enrolled on the PlanB trial, and found that lobular tumors were associated with higher tumor and nodal stages, lower grade, and lower RS than non-lobular tumors, but similar disease-free survival (DFS). In contrast to our findings, Christgen et al. found that Grade 3 disease was associated with poorer DFS in lobular tumors, but high RS was not^20^.

Two studies have used SEER data to evaluate RS in BCA subtypes other than IDC. Wang et al. evaluated RS in 83,665 patients with N0 ER + BCA measuring under 5 cm, and belonging to one of eight different tumor subtypes (IDC, ILC, DLC, cribriform, tubular, mucinous, micropapillary, and intraductal papillary adenocarcinoma with invasion). They reported that IDC was more likely to have RS > 30 than IDC and DLC, but that IDC, ILC, and DLC had similar mean RS (which were higher than the mean RS of the other subtypes evaluated). RS was prognostic for breast cancer specific survival (BCSS) in IDC, ILC, and DLC, but not in other tumor subtypes^21^. Kizy et al. used SEER data to evaluate the prognostic and predictive impact of RS in Stage I to III ER + ILC (*n* = 7316), and similar to us, found that high RS was prognostic for worse OS, but did not predict chemotherapy benefit in ILC^18^. These studies are limited by the use of SEER data, which does not record patient comorbidities, collects incomplete information on chemotherapy and endocrine therapy use^22^, and did not collect HER2 status until 2010^18^. In addition, Kizy’s study included patients with Stage III disease, for whom RS is not recommended by national clinical practice guidelines^23,24^, while Wang’s study used the initial cutpoints for intermediate and high RS^10^, which have now been largely replaced by the TAILORx cutpoints^25^.

Investigators have also evaluated other gene expression assays in ILC. Beumer et al. evaluated the prognostic value of MammaPrint in 217 ILC cases treated on one of five clinical trials, and found that high-risk MammaPrint was an independent poor prognostic factor for distant metastasis-free survival (DMFS), distant metastasis-free interval, and OS in ILC^26^. Metzger et al. compared clinicopathologic risk factors and MammaPrint results in IDC (*n* = 4826) and ILC (*n* = 487) patients enrolled on the MINDACT trial, and found that IDC and ILC had similar clinical risk distributions, but ILC was less likely than IDC to be genomically high risk. DMFS and DFS rates for IDC and ILC of similar genomic risk were similar to each other, suggesting prognostic value for MammaPrint in ILC^27^. The patient population for both of these studies was heterogeneous, and included ER-negative and HER2-positive cases^26,27^. Sestak et al. assessed the prognostic value of the EndoPredict assay in a cohort of 470 postmenopausal women with N0 and N + ILC treated with endocrine therapy on the ATAC, ABCSG-6, or ABCSG-8 trials. EPClin was prognostic for 10-year distant recurrence (DR) in ILC, and DR rates were similar in IDC and ILC patients with similar genomic risks by EpClin^28^. In contrast, Laenkholm compared results of the PAM50 assay in a Danish cohort of postmenopausal women with N0 or N1 ER + HER2-negative ILC (*n* = 340) and IDC (*n* = 1570), and found that ILC patients had significantly poorer 10-year DR than IDC patients with similar ROR scores^29^.

A different spectrum of genetic alterations in ILC compared with IDC may underlie the differing biologic behavior of the two histologies. In addition to the pathognomonic inactivation of *CDH1*, mutations in *FOXA1* and in *PIK3CA, PTEN and AKT* are more common in ILC than in IDC, and *GATA3* mutations are more frequent in IDC^4,30–32^. ILC is also associated with increased gains in chromosome 1q, 8q, and 16p; losses of 8p23-p21, 11q14.1-q25, and 16q; and amplifications of 1q32, 8p12, and 11q13 compared with IDC^4,30,32,33^. Thus, lobular-specific molecular assays may improve prognostication in ILC over currently available gene expression assays. LobSig, an ILC-specific 194-gene signature that incorporates gene expression and copy number, has recently been developed, and showed better prognostic ability in ILC than ROR and RS in a stepwise, multivariate Cox proportional hazards model^4,30^. Further studies to validate lobular-specific assays such as LobSig are warranted.

Our finding that the OS benefit of adjuvant chemotherapy is seen only in IDC warrants further study, especially as this finding may have been due to insufficient statistical power to detect benefit in less common BCA subtypes. While ILC is typically associated with both lower RS^14–16^ and poorer response to chemotherapy^17,19^, the trials that established that high RS is predictive of chemotherapy benefit in ER + HER2-negative BCA did not differentiate between tumor subtypes^8,11,12^, and no prospective trial has evaluated chemotherapy in ILC alone. Evaluation of histology, RS, and chemotherapy benefit in patients enrolled on large prospective clinical trials, such as TAILORx, may help further clarify this finding.

Our study has several strengths and limitations, due to use of NCDB data. Strengths include the large sample size, utilizing real-world data from CoC-accredited institutions. Limitations include the retrospective nature of the study, lack of information on cancer recurrence, relatively short duration of follow-up, and lack of central pathologic review.

In summary, our study confirmed that ILC and mixed BCA histologies are associated with distinct clinicopathologic and prognostic features compared with IDC, and with lower RS. Despite this, RS remains prognostic for OS in ILC and DLC. Further studies are warranted to evaluate our finding that chemotherapy benefit is limited to IDC.

## Methods

### Case selection

We utilized a dataset derived from the 2005-2016 National Cancer Database (NCDB). NCDB is a nationwide, facility-based database jointly sponsored by the American Cancer Society and the American College of Surgeons Commission on Cancer (CoC). The NCDB contains data collected on over 34,000,000 cancer cases from over 1500 CoC-accredited hospitals, representing over 70% of newly diagnosed cancers^34^, and 80% of newly diagnosed breast cancers in the United States^35^. The NCDB Participant User File (PUF) is a HIPAA-compliant data file, which is made available to investigators from CoC-accredited cancer programs who complete an application process. The data used in the study are derived from a de-identified NCDB file. The American College of Surgeons and the Commission on Cancer have not verified and are not responsible for the analytic or statistical methodology employed, or the conclusions drawn from these data by the investigator. The NCDB PUF contains de-identified patient data, including demographic information, tumor site and pathology data, first course of treatment, and mortality. The NCDB PUF does not contain information on recurrence. The NCDB began collecting information on results of gene expression assays in 2010.

The study population included women age 75 and under diagnosed between 1/1/2010 and 1/1/2014 with estrogen receptor-positive (ER+) HER2- BCA, measuring up to 5 cm, with 0–3 pathologically involved axillary nodes, treated with definitive surgery as first treatment, with tumor that contained ductal or lobular histology, and with numeric RS available. Tumor subtypes were coded as IDC, ILC, infiltrating duct and lobular carcinoma, infiltrating duct mixed with other types of carcinoma (DOC), and infiltrating lobular mixed with other types of carcinoma (LOC). Demographic information obtained included age at diagnosis, race, and ethnicity, as well as estimated annual household income, rurality and educational attainment of area of residence. Clinical characteristics included tumor size in mm, histologic grade, axillary node involvement, Charlson-Deyo comorbidity index, and numeric RS. Micrometastatic nodal involvement (pN1mi) was classified as node positive. RS was characterized as low, intermediate, or high using TAILORx cutpoints, where 0–10 was defined as low, 11–25 as intermediate, and 26–100 as high.

### Statistical analysis

Associations between categorical variables were examined using the chi-square test. The Cox proportional hazards model was used to examine the difference in OS between histologic subtypes while controlling for age, race, ethnicity, Charlson-Deyo comorbidity index, median estimated annual household income of area of residence, rurality of area of residence, and educational attainment of area of residence, RS, tumor size, grade, node involvement and treatment. The estimated HR for each variable in the model, along with its 95% CI, was reported. All tests were two-sided with significance level <5%. Proportionality assumption was examined, and no violation was detected. All analyses were conducted using SAS 9.4 (SAS Institute Inc., Cary, NC, USA).

### Ethics

NCDB PUF data are de-identified, and compliant with HIPAA. Hospitals, health care providers, and patients are not identified. Patient informed consent is not obtained prior to institutional data submission to NCDB. As this study utilizes de-identified patient data, with no attempt made to contact or re-identify the subjects, it is deemed exempt from oversight by the Institutional Review Board of Albert Einstein College of Medicine.

### Reporting summary

Further information on research design is available in the Nature Research Reporting Summary linked to this article.

## Supplementary information

Reporting Summary

## Data Availability

The NCDB PUF is a HIPAA-compliant data file, which is made available to investigators from CoC-accredited cancer programs who complete an application process.
