# Supplementary material for: The 21-gene recurrence score in early non-ductal breast cancer: a National Cancer Database analysis
Source: NPJ Breast Cancer. 2022 Jan 13;8:4. doi: 10.1038/s41523-021-00368-9 (PMC8758772; doi:10.1038/s41523-021-00368-9)
Supplement: Supplementary file 1 — Reporting Summary [file 41523_2021_368_MOESM1_ESM.pdf]

nature research

Corresponding author(s): NPJBCANCER-01391R Makower

Last updated by author(s): Nov 14, 2021

Reporting Summary

Nature Research wishes to improve the reproducibility of the work that we publish. This form provides structure for consistency and transparency in reporting. For further information on Nature Research policies, see our Editorial Policies and the Editorial Policy Checklist.

Statistics

For all statistical analyses, confirm that the following items are present in the figure legend, table legend, main text, or Methods section.

n/a	Confirmed	
☐	☒	The exact sample size (*n*) for each experimental group/condition, given as a discrete number and unit of measurement
☐	☒	A statement on whether measurements were taken from distinct samples or whether the same sample was measured repeatedly
☐	☒	The statistical test(s) used AND whether they are one- or two-sided<br>*Only common tests should be described solely by name; describe more complex techniques in the Methods section.*
☐	☒	A description of all covariates tested
☐	☒	A description of any assumptions or corrections, such as tests of normality and adjustment for multiple comparisons
☐	☒	A full description of the statistical parameters including central tendency (e.g. means) or other basic estimates (e.g. regression coefficient) AND variation (e.g. standard deviation) or associated estimates of uncertainty (e.g. confidence intervals)
☐	☒	For null hypothesis testing, the test statistic (e.g. *F*, *t*, *r*) with confidence intervals, effect sizes, degrees of freedom and *P* value noted<br>*Give P values as exact values whenever suitable.*
☒	☐	For Bayesian analysis, information on the choice of priors and Markov chain Monte Carlo settings
☒	☐	For hierarchical and complex designs, identification of the appropriate level for tests and full reporting of outcomes
☒	☐	Estimates of effect sizes (e.g. Cohen's *d*, Pearson's *r*), indicating how they were calculated

Our web collection on statistics for biologists contains articles on many of the points above.

Software and code

Policy information about availability of computer code

Data collection	download from NCDB website
Data analysis	SAS 9.4 was used and the code is available upon request.

For manuscripts utilizing custom algorithms or software that are central to the research but not yet described in published literature, software must be made available to editors and reviewers. We strongly encourage code deposition in a community repository (e.g. GitHub). See the Nature Research guidelines for submitting code & software for further information.

Data

Policy information about availability of data

All manuscripts must include a data availability statement. This statement should provide the following information, where applicable:

- Accession codes, unique identifiers, or web links for publicly available datasets
- A list of figures that have associated raw data
- A description of any restrictions on data availability

The NCDB PUF is a HIPAA-compliant data file, which is made available to investigators from CoC-accredited cancer programs who complete an application process.

Field-specific reporting

Please select the one below that is the best fit for your research. If you are not sure, read the appropriate sections before making your selection.

☒ Life sciences ☐ Behavioural & social sciences ☐ Ecological, evolutionary & environmental sciences

For a reference copy of the document with all sections, see nature.com/documents/nr-reporting-summary-flat.pdf

Life sciences study design

All studies must disclose on these points even when the disclosure is negative.

Sample size	The study population included women age 75 and under diagnosed between 1/1/2010 and 1/1/2014 with estrogen receptor-positive (ER+) HER2- BCA, measuring up to 5 cm, with 0-3 pathologically involved axillary nodes, treated with definitive surgery as first treatment, and with numeric RS available.
Data exclusions	RS non available were not included in the primary analysis
Replication	Proportional hazard assumption checking. Plotted the Schoenfeld residuals against survival time for the exposures of cox regression we did. No major violation found.
Randomization	Retrospective study of existing data
Blinding	NA

Reporting for specific materials, systems and methods

We require information from authors about some types of materials, experimental systems and methods used in many studies. Here, indicate whether each material, system or method listed is relevant to your study. If you are not sure if a list item applies to your research, read the appropriate section before selecting a response.

Materials & experimental systems

n/a	Involved in the study	
☒	☐	Antibodies
☒	☐	Eukaryotic cell lines
☒	☐	Palaeontology and archaeology
☒	☐	Animals and other organisms
☒	☐	Human research participants
☐	☒	Clinical data
☒	☐	Dual use research of concern

Methods

n/a	Involved in the study	
☒	☐	ChIP-seq
☒	☐	Flow cytometry
☒	☐	MRI-based neuroimaging

Clinical data

Policy information about clinical studies

All manuscripts should comply with the ICMJE guidelines for publication of clinical research and a completed CONSORT checklist must be included with all submissions.

Clinical trial registration	NA - retrospective NCDB data
Study protocol	Retrospective NCDB data - IRB exempt
Data collection	Retrospective study of existing data that had been collected from CoC accredited hospitals
Outcomes	Data was submitted by CoC accredited hospitals and collected by NCDB. Associations between categorical variables were examined using the chi-square test. The Cox proportional hazards model was used to examine the difference in overall survival (OS) between histologic subtypes while controlling for age, race, ethnicity, Charlson-Deyo comorbidity index, median estimated annual household income of area of residence, rurality of area of residence, and educational attainment of area of residence, RS, tumor size, grade, node involvement and treatment. The estimated HR for each variable in the model, along with its 95% CI, was reported. All tests were two-sided with significance level <5%.
